# COVID – 19 in two dialysis centers situated in two neighbouring states of the Western Balkans

**DOI:** 10.1186/s12882-023-03080-x

**Published:** 2023-02-18

**Authors:** Enisa Mesic, Mirna Aleckovic-Halilovic, Karolina Paunovic, Alexander Woywodt, Mirha Pjanic, Goran Paunovic

**Affiliations:** 1 Department of Nephrology, Dialysis and Transplantation, University Hospital Tuzla, Ulica Prof. Dr. Ibre Pašića, 75000 Tuzla, Bosnia and Herzegovina; 2 Nephrology Department, Niš Clinical Centre, Niš, Serbia; 3 Department of Renal Medicine, Lancashire Teaching Hospitals NHS Foundation Trust, Preston, Lancashire UK

**Keywords:** COVID-19, Western Balkans, Infection control, Health-system emergency preparedness

## Abstract

**Background:**

Bosnia and Herzegovina (BiH) and Serbia are countries in the Western Balkans that share parts of their social and political legacy from the former Yugoslavia, such as their health care system and the fact that they are not members of European Union. There are very scarce data on COVID – 19 pandemic from this region when compared to other parts of the world and even less is known about its impact on the provision of renal care or differences between countries in the Western Balkans.

**Materials and methods:**

This observational prospective study was conducted in two regional renal centres in BiH and Serbia, during the COVID – 19 pandemic. We obtained demographic and epidemiological data, clinical course and outcomes of dialysis and transplant patients with COVID – 19 in both units. Data were collected a via questionnaire for two consecutive time periods: February – June 2020 with a total number of 767 dialysis and transplant patients in the two centres, and July – December 2020 with a total number of 749 studied patients, corresponding to two of the largest waves of the pandemic in our region. Departmental policies and infection control measures in both units were also recorded and compared.

**Results:**

For a period of 11 months, from February to December 2020, 82 patients on in-centre haemodialysis (ICHD), 11 peritoneal dialysis patients and 25 transplant patients who tested positive for COVID-19. In the first study period, the incidence of COVID – 19 positive in Tuzla was 1.3% among ICHD patients, and there were no positive peritoneal dialysis patients, or any transplant patients who tested positive. The incidence of COVID-19 was significantly higher in both centres in the second time period, which corresponds to the incidence in general population. Total deaths of COVID-19 positive patients was 0% in Tuzla and 45.5% in Niš during first, and 16.7% in Tuzla and 23.4% in Niš during the second period. There were notable differences in the national and local/departmental approach to the pandemic between the two centres.

**Conclusion:**

There was poor survival overall when compared to other regions of Europe. We suggest that this reflects the lack of preparedness of both of our medical systems for such situations. In addition, we describe important differences in outcome between the two centres. We emphasize the importance of preventative measures and infection control and highlight the importance of preparedness.

## Introduction

There is a substantial body of literature describing the COVID 19 pandemic in Western Europe and also delineating its impact on the provision of renal care [1]. In comparison, very little attention has been paid to central and eastern Europe [2]. In the Western Balkans countries, nephrologists have faced significant challenges even before the pandemic be it through lack of funding, or due to the impact of difficult political circumstances. Despite those challenges some Eastern countries started implementing infection control measures before their Western counterparts and adherence to government guidance may also be better, for example when people feel more vulnerable or as a result of a longstanding culture of obeying government guidance generally [3]. Here, we report on the impact of COVID-19 on dialysis and transplant patients during two waves of the pandemic in two renal centres in BiH and Serbia. We provide a narrative review of these two centres’ experience, describe incidence and mortality of COVID-10 in renal patients and highlight differences and similarities between the two centres.

## Materials and methods

### Study setting

This observational prospective study was conducted in two clinical centres in Bosnia and Herzegovina (BiH) and Serbia, both countries from the Western Balkans region. The total population of BiH is estimated at 3,531,000, with a prevalence of established renal failure estimated at 2557 (724 per million population – pmp), of which 2068 receiving chronic haemodialysis treatment, 97 patients on peritoneal dialysis, and 392 kidney transplant patients (2020 data). There are 29 dialysis centres in BiH. The 25-bed dialysis centre in Tuzla is part of University Hospital and treats 170 chronic dialysis patients from the city of Tuzla with a population of around 120.000 and the surrounding areas [4]. It is also a transplanting centre. The population of Serbia is estimated at 6,899,000, with a prevalence of established kidney failure of 5516 (888 pmp), of which 4221 receiving chronic haemodialysis treatment, 457 patients on peritoneal dialysis, and 832 kidney transplant patients (2020 data). There are 64 dialysis centres in the Republic of Serbia. The dialysis centre in Niš is part of Niš Clinical Centre, a state-owned University hospital. It serves the city of Niš with a population around 190.000 and surrounding areas in South and South Eastern Serbia and treats around 264 chronic dialysis patients [5]. The majority of patients treated in both centres are adults. Haemodialysis, haemodiafiltration and peritoneal dialysis are available in both centres for outpatients, inpatients, and management of acute kidney injury, for usual indications.

## Methods

The following variables were collected and monitored throughout the study period: demographic and epidemiological data (patient population, doctor/nurse per patient ratio, incidence of COVID-19 among patients and healthcare staff, outcomes and mortality), COVID – 19 preventive and control measures in dialysis centres, availability of face masks for patients, availability and quality of personal protective equipment (PPE) for healthcare workers, spatial and technological requirements for isolation in dialysis facilities, triage and transport to and from dialysis facilities, adequacy of dialysis units staffing, modification of haemodialysis treatment time, disinfection procedures and frequency of testing for COVID – 19. Patients with COVID – 19 and receiving haemodialysis or peritoneal dialysis and who had a renal transplant were analysed, with special interest in clinical course and outcomes. Severity of disease was judged upon initial patient’s presentation as following: patients with mild COVID-19 disease were the ones who wouldn't require hospitalisation at Clinic for Infectious Diseases if there had been enough capacity and personnel to organize separate regular outpatient dialysis sessions for these patients. Patients with moderate COVID-19 disease were the ones who required hospitalization at Clinic for Infectious Diseases due to their symptoms, however, they were stable and didn't require admission to the ICU. Patients with severe form of COVID-19 disease were the ones who required admission to the ICU. Data were collected by filling out a questionnaire once a month for 11 months (February – December 2020) and recorded into database. Data were analysed and compared for two consecutive time periods: February – June 2020 with total number of 767 studied patients, and July – December 2020 with total number of 749 studied patients. Chi-squared test was used in the analysis of differences in the patient distribution, number of patients and healthcare workers with COVID-19 infection between the two centres in the two study periods. During these two time periods, epidemiological situation in BiH and Serbia was significantly different.

## Results

### Patient population and resources in both centres

Table 1 shows differences in patient population and resources during the two study periods. Of note, Niš had a higher number of patients receiving in-centre chronic haemodialysis (ICHD) and more than twice the number of patients receiving continuous ambulatory peritoneal dialysis (CAPD) compared to Tuzla, but not significantly different (*p* < 0.26, *p* < 0.89, respectively). The number of kidney transplant patients was similar in both centres. The difference in the number of dialysis patients corresponded to the difference in population size in these two regions (Niš had population of 190,000 inhabitants, and Tuzla about 120,000 inhabitants) [4, 5]. In the first study period, the total number of patients treated with some method of the renal replacement therapy (RRT) was 767, and in the second period 749. Doctor-per-patient and nurse-to-patient ratios were approximately the same in both centres (Table 1). Unfortunately, we were not able to collect other demographic data such as age and sex of patients, due to the limited staffing and other limited resources in both centres during these periods.

**Table 1:** Number of doctors, nurses, dialysis and kidney transplant patients in dialysis centres in Tuzla and Niš

	Tuzla February – June 2020	Niš February – June 2020	Total number	Tuzla July – December 2020	Niš July – December 2020	Total number
ICHD patients	157	217	374	147	206	353
CAPD patients	23	57	80	23	58	81
Kidney transplant patients	160	153	313	164	151	315
Total number	340	427	767	334	415	749
Doctor-per-patient ratio	1:52	1:43		1:50	1:41	
Nurse-to-patient ratio (during one dialysis shift)	1:6	1:7		1:6	1:8	

*Abbreviations*: *ICHD* In-centre haemodialysis, *CAPD* Continuous Ambulatory Peritoneal Dialysis

### Infection control measures

Table 2 summarises implemented measures related to usage of PPE, spatial conditions, transport to and from dialysis facilities, and COVID – 19 screening in dialysis units in Tuzla and Niš. Infection control measures did not change significantly between the two study periods. In both centres there was a separate area for dialysis of COVID – 19 positive patients, but without sufficient physical distance, and also without separate transportation to outpatient dialysis for COVID – 19 positive patients. Of note, better quality PPE for staff and patients was available at the dialysis centre in Niš compared to Tuzla. Also, medical staff of dialysis facility in Niš underwent regular staff surveillance testing for COVID – 19 more often compared to Tuzla. In some instances insufficient supply of PPE led to a situation where patients and also staff had to use their masks for several days and washed and ironed their masks at home in between use. However, patient triage was done in a similar manner in both centres except for the fact that Tuzla implemented telephone reporting of COVID – 19 symptoms or suspicious contacts to the dialysis unit. Also, suspected cases who met epidemiological or clinical criteria for COVID – 19 in dialysis facility in Tuzla, were dialysed in separate areas, despite of negative polymerase chain reaction (PCR) testing for COVID-19. Duration of dialysis and disinfection were performed in a very similar manner (Table 2). The Tuzla centre also noted a case where three dialysis patients were thought to have been infected during transport to dialysis after the ambulance driver later tested COVID-19 positive. Remarkably, with immediate moving of the entire group of patients (20 pts) to the night shift of dialysis, there was no further spread of the infection.

**Table 2:** Usage of PPE, spatial conditions, transport to and from dialysis facilities, COVID-19 screening, patient triage, dialysis length, and disinfection in dialysis units Tuzla and Niš

	Tuzla February – December 2020	Niš February – December 2020
Total number of chronic dialysis patients (ICHD + CAPD)	180	275
Regular surveillance testing of staff for COVID-19 (overall total number of testing per center)	1 X (PCR)	7 X (PCR; rapid antigen test)
Face masks for staff in dialysis unit	Surgical	KN95
Other protective equipment for staff (face shields, surgical caps, shoe covers, long sleeved gowns)	Only for suspicious and positive patients	Every day for dealing with all patients
Gloves	Obligatory	Obligatory
Distance of 2 m between the dialysis beds	No	No
Separate transportation of COVID-19 dialysis patients	No	No
Surveillance testing of dialysis patients for COVID-19 (type of test)	1 x (PCR)	No
Face masks for dialysis patients	Cloth or Surgical	Surgical
Patients' education about fever or other symptoms of COVID-19 and self-reporting	Yes	Yes
Patients' self reporting of symptoms and/or contact with someone who has COVID-19 by telephone before travelling to the dialysis unit	Yes	No
Patients' triage on entry to dialysis unit	Yes	Yes
Shortening dialysis time to allow adequate time for disinfection	Yes; 3:45 h	Yes; 3:45 h
Area disinfection after each dialysis session	Yes	Yes

*Abbreviations*: *PPE* Personal Protective Equipment, *ICHD* In-centre haemodialysis, *CAPD* Continuous Ambulatory Peritoneal Dialysis

### Incidence of COVID

For a period of 11 months, from February to December 2020, there were 82 ICHD patients, 11 CAPD patients and 25 kidney transplant patients who tested positive for COVID-19 among these two dialysis facilities (Table 3). In the first study period, the incidence of COVID – 19 in Tuzla was 1.3% among ICHD patients, and there were no positive CAPD patients, nor patients with kidney graft. The incidence of COVID – 19 positive ICHD patients in Niš was 7.4%, and unlike in Tuzla, there were also COVID – 19 positive CAPD (5.3%) and kidney transplant patients (1.96%). The incidence of COVID-19 was higher in both centres during the second study period, not only for ICHD patients, but also for CAPD and kidney transplant patients.

**Table 3:** ICHD, CAPD and kidney transplant patients and staff with COVID-19 in 2020

Centre Period	Tuzla February to June	Niš February to June	Tuzla July to December	Niš July to December
	N (%)	N (%)	N (%)	N (%)
ICHD patients with COVID-19	2/157 (1.3)	16/217 (7.4)	16/147 (10.9)	48/206 (23.3)
CAPD patients with COVID-19	0/23	3/57 (5.3)	4/23 (17.4)	4/58 (6.9)
Kidney transplant patients with COVID-19	0/160	3/153 (1.96)	10/164 (6.1)	12/151 (7.9)
Total COVID-19 positive patients	2/340 (0.6)	22/427 (5.2)	30/334 (9.0)	64/415 (15.4)
Doctors with COVID-19	0/9	6/11 (54.5)	3/9 (33.3)	3/11 (27.3)
Nurses with COVID-19	0/24	14/39 (35.9)	5/24 (20.8)	17/39 (43.6)

*Abbreviations*: *ICHD* In-centre haemodialysis, *CAPD* Continuous Ambulatory Peritoneal Dialysis

There were some differences between the two centres (Table 3). Apart from CAPD patients, Tuzla had lower number of COVID-19 positive patients in both time periods, especially in the first study period when only two (out of 157) COVID-19 positive ICHD patients were registered and no CAPD and kidney transplant patients. Overall number of COVID-19 positive ICHD patients was significantly lower in Tuzla compared to Niš in both study periods (*p* = 0.0065, *p* = 0.0028, respectively). Also, Tuzla had significantly lower number of doctors and nurses with COVID-19 compared to Niš in the first study period (*p* = 0.008, *p* = 0.0009, respectively).

### Outcomes and mortality

During the first study period in dialysis centre in Niš, among all COVID-19 positive ICHD patients (16), there were six patients with moderate (37.5%) and five patients with severe disease (31.25%), and a total of 10 patients were admitted to Intensive Care. At the same time in dialysis centre in Tuzla, there were two COVID-19 positive ICHD patients of which one was asymptomatic and the other had a moderate disease. During the second study period, most patients had more severe forms of the disease. In dialysis centre in Niš, among all COVID-19 positive ICHD patients (48), 30 patients had moderate disease (62.5%) and 11 patients had more severe disease (22.9%). Thirty patients were hospitalized in ICUs. During that time, in Tuzla, out of a total of 16 COVID-19 positive ICHD patients, seven had moderate clinical symptoms (43.75%) and four had a severe disease (25%). None of the patients was transferred into the ICU. In our study, hospitalization was reported in 96.5% (83/86) of patients in the dialysis centre in Niš and 50% (16/32) in the dialysis centre in Tuzla. Among COVID-19 positive CAPD patients in Niš, all three patients in the first study period and four patients in the second study period had a severe form of disease. In Tuzla, in the first study period, there were no COVID-19 positive CAPD patients, while three patients were positive in the second period, with a mild disease and one with moderate symptoms requiring hospital treatment. All three COVID-19 positive kidney transplant patients in Niš had a severe form of disease in the first study period. In the second study period, there were four patients with mild disease, seven patients with moderate and one patient with severe form of disease. Eleven patients needed hospitalization. In Tuzla, no COVID-19 positive kidney transplant patients were registered in the first study period, and in the second study period, of the ten positive patients, one patient had severe form of disease and died, two patients had moderate disease requiring hospital treatment, and rest of the patients had mild symptoms. Table 4 provides mortality according to the time period, type of treatment and dialysis facility. The overall mortality of COVID-19 negative ICHD patients in the Tuzla dialysis facility during 2020 was 21.08% (31 out of 147 patients died), and the overall mortality of ICHD patients during 2020, with and without COVID-19 infection, was 23.08% (35/147). The overall mortality of COVID-19 negative ICHD patients in the dialysis facility in Niš during 2020 was 24.3% (50 out of 206 patients died), and the overall mortality of ICHD patients in 2020, with or without COVID-19 infection, was 35.4% (73/206).

**Table 4:** Mortality of ICHD, CAPD and kidney transplant patients with COVID-19 in 2020

Centre Period	Tuzla February to June	Niš February to June	Tuzla July to December	Niš July to December
	N (%)	N (%)	N (%)	N (%)
Deceased ICHD patients with COVID-19	0/2	5/16 (31.3)	4/16 (25.0)	12/48 (25.0)
Deceased CAPD patients with COVID-19	0/0	3/3 (100.0)	0/4 (0.0)	3/4 (75.0)
Deceased kidney transplant patients with COVID-19	0/0	2/3 (66.67)	1/10 (10.0)	0/12
Total deaths of COVID-19 positive patients	0/2	10/22 (45.5)	5/30 (16.7)	15/64 (23.4)

*Abbreviations*: *ICHD* In-centre haemodialysis, *CAPD* Continuous Ambulatory Peritoneal Dialysis

## Discussion

Bosnia and Herzegovina and Serbia are countries in the Western Balkans that have emerged from the former Yugoslavia and therefore share aspects of the organisation and structure of their health care systems. Another similarity is a strong culture of state-delivered public health and public adherence to guidance issued through this route. The impact of the COVID-19 pandemic on countries in Eastern Europe has only recently received some attention. It remains unclear how some of the Central and Eastern European countries managed to keep the number of cases low in the first wave of the pandemic [2]. However, by mid-March 2021, six of the 10 countries with the highest mortality rates per 100,000 inhabitants were from Central and Eastern Europe [2]. Stark differences also exist in the prevalence of chronic kidney disease between Western and Eastern Europe [6]. These may be partly explained by a higher prevalence of diabetes, hypertension, obesity and tobacco use [6]. Surprisingly little is known about the impact of COVID-19 on the provision of care in nephrology in these countries.

Published data on the incidence of COVID-19 in the dialysis population in other parts of Europe, USA, Canada, China and Ecuador, demonstrates considerable variation of the reported incidence ranging from 2.5% to 23.2% [7–20]. Here, we report data from the Western Balkan on a background of very scarce or non-existent data in the region overall. Remarkably, despite our overall circumstances, the overall incidence was therefore not as high as reported in other, more developed countries. In Tuzla, the overall incidence of COVID-19 positive RRT patients during 11 months in both studied periods was 9.58% (32 out of 334) patients, and in Niš 20.72% (86 out of 415).

Our study showed that in both centres, in the first time period, from February to the end of June 2020, the incidence of COVID-19 positive ICHD patients was significantly lower (4.81%) than in the second time period, from July to the end of December 2020 (18.13%). These data correspond to the incidence of COVID – 19 in the general population of BiH and Serbia: During the first time period i.e. during June 2020 the 7-day average incidence of COVID – 19 cases was 124 pmp in BiH and 257 pmp in Serbia [21, 22]. At the end of June 2020, 4453 COVID-19 positive patients (0.136%) were reported in BiH, and 14,564 COVID-19 positive patients (0.167%) in Serbia [21, 22]. The situation changed significantly during the autumn wave of the pandemic in October 2020. During this period the 7-day average incidence of new COVID-19 cases was 1516 pmp in BiH and 1726 pmp in Serbia, while in December 2020 a total of 110,985 patients were reported in BiH (3.39%) and 337,923 patients in Serbia (3.87%) [21, 22]. Of note, the second time period featured a relatively quick cessation of lock-down restrictions as well as free entry to both our countries whereas a complete lockdown in BiH and Serbia had been in force during the first period. No accurate data are available on how many patients were placed in intensive care units but we estimate this to be around 45% of hospitalized patients in both centres. Of note, in Tuzla, we were forced to hospitalize all COVID19 positive chronic haemodialysis patients regardless of their clinical status since we did not have enough capacity and personnel to organize separate regular outpatient dialysis sessions for these patients. In our study mortality for ICHD patients with COVID19 for Tuzla was zero in the first wave, and 25% in the second wave, while for Niš it was 31,3% and 25%, respectively. The overall mortality for patients with RRT (ICHD, CAPD and renal graft) for Tuzla was zero in the first and 16,7% for the second wave, and for Niš it was 45,5% and 23,44%, respectively. In comparison in ERACODA the 28-day probability of death was 21.3% (95% confidence interval 14.3–30.2%) in transplant patients and 25% (95% CI 20.2–30%) in dialysis patients that went up to 33.5% for hospitalized dialysis patients [23]. For comparison a multicentric French study of 2336 patients reported that 81% of patients were hospitalized and 28% of patients died whereas others have reported mortality rates between 10 and 43% [8–10, 12, 24–26]. If we compare the overall mortality of ICHD patients in Tuzla during 2020, with and without COVID-19 infection (23.8%; 35/147) with mortality in 2019, when 50 of 156 patients died (32%), we conclude that COVID-19 in the Tuzla dialysis facility during 2020 did not increase the mortality of ICHD patients [27]. The overall mortality of ICHD patients in Niš during 2020, with or without COVID-19 infection, was 35.44% (73/206). In 2019, 50 ICHD patients (or 23.04%; 50/217) died in the dialysis facility in Niš [28].

Of note, national recommendations in both countries diverged most of the time. As an example, restrictions were significantly eased during Orthodox Easter and Christmas and during elections in Serbia and during Eid in BiH, which was promptly followed by an increase in COVID-19 cases in both countries (data not shown).

Both centres put a lot of resources into infection control measures and into education of staff and patients as one of the few strategies available to us. Our efforts were hampered by lack of adequate PPE and the absence of an organised effort for vaccination for anyone except for medical staff. The Tuzla centre did not have the capacity to ensure minimum distances or mechanical barriers between HD patients, and we did not have sufficient isolation rooms, or separate transport either. The Tuzla centre therefore had to hospitalize all COVID positive HD patients and decided to reorganize night shifts for cohorting suspected cases (symptomatic patients who were PCR negative as well as patients with positive close contacts) and those discharged from COVID wards. Hospitalization per se could be attributing factor for high mortality especially in frail patients [29]. In ERACODA, it was also noted that mortality was very low in dialysis patients who were not hospitalized [23].

In comparing infection control measures between the two centres (Table 2) it is also noteworthy that Niš had better protective equipment, namely KN95 face masks for staff and surgical masks for patients while staff in Tuzla had only surgical masks and patients were wearing cloth masks. An Irish study emphasized the importance of wearing masks in dialysis centres [30]. Remarkably, there is no national authority in BiH that would issue or coordinate such regulations but mandatory wearing of masks for all patients and staff was introduced in the Tuzla Dialysis Centre in January 2020, together with formal triage before entering the Dialysis Centre in February 2020. These measures may have contributed to the low incidence and mortality seen in Tuzla during the first wave. It is difficult to compare infection control measures to those taken elsewhere in our region. One report from Slovenia described better PPE, a more favourable nurse-to-patient ratio and distance between dialysis beds but also noted the lack of individualised transport for COVID-19 positive patients [31]. Finally, lower rates of staff infection in Tuzla may be due to the fact than in the centre in Niš had surveillance testing for COVID19 for their staff, which Tuzla did not.

There were other important differences between the two centres. The centre in Niš had access to a number of mobile reverse osmosis devices which facilitated dialysis of acute patients at several isolated locations (COVID hospitals). In Tuzla, there were only 4 mobile dialysis machines for all acute patients. This shortage led to a situation where continuous RRT methods could not be offered any longer and there were no funds to purchase additional equipment. This situation was further compounded by shortage of trained dialysis nurses which may explain the very poor outcome in patients with acute kidney injury (AKI) and COVID-19, who had to be dialyzed.

The COVID-19 pandemic has affected many different countries with different circumstances globally and an ideal approach to fighting infection does not exist. Flexibility of the strategy is key to preventing the spread of infection and timely planning can overcome some the effects of the pandemic [32, 33]. Another area that also deserves consideration is pan-European cooperation and support which has worked so well in other areas for example during the earthquakes in Turkey and Armenia [34]. We suggest that the European Renal community considers ways in which nephrologists in affluent well developed European countries can help their counterparts in less well developed countries in situation like this.

## Conclusion

We describe the impact of COVID-19 on the provision of renal care in two countries in the Western Balkans. Little is known about the overall effects of the pandemic on health care in this region and even less about the impact on patients with kidney disease and their care providers. We report poor outcome especially in patients with AKI and COVID when compared to other European countries. We emphasize the unpreparedness of both our medical systems for situations like this. Moreover, the pandemic affected two health care systems that were underfunded long before the pandemic. Lack of availability of diagnostic tools, drugs and vaccines in both countries represented a particular challenge. Our experience highlights the issues of SARS-CoV-2 in developing countries and countries with significant political challenges such as Serbia and BiH. We also emphasise the importance of simple interventions and note that the spread of SARS-CoV-2 within dialysis centres, even in resource-depleted countries, can be slowed down using relatively cheap infection control measures. Finally, we suggest that the pandemic is an issue that transcends borders and political differences and therefore requires a pan-European and collaborative approach [2].

## Data Availability

The data underlying the manuscript “COVID – 19 in two dialysis centers situated in two neighbouring states of the Western Balkans” will be shared on reasonable request to the corresponding author.
